# Nanobiotechnology Unveils the Power of Probiotics: A Comprehensive Review on the Synergistic Role of Probiotics and Advanced Nanotechnology in Enhancing Geriatric Health.

**DOI:** 10.7759/cureus.80478

**Published:** 2025-03-12

**Authors:** Onkar Kadam, Swayamprava Dalai, Bhawna Chauhan, Rashmi Ranjan Guru, Subhodip Mitra, Namita Raytekar, Rahul Kumar

**Affiliations:** 1 Biotechnology, Symbiosis Centre for Nanoscience and Nanotechnology, Symbiosis International (Deemed) University, Pune, IND; 2 School of Biotech Engineering and Food Technology, Chandigarh University, Chandigarh, IND; 3 Hospital Administration, All India Institute of Medical Sciences, Jodhpur, Jodhpur, IND; 4 Hospital Administration, Postgraduate Institute of Medical Education and Research, Chandigarh, Chandigarh, IND; 5 Hospital Administration, All India Institute of Medical Sciences, Kalyani, Kolkata, IND; 6 Medical Technology, Symbiosis Institute of Health Sciences, Pune, IND; 7 Hospital Administration, Symbiosis University Hospital & Research Centre, Pune, IND

**Keywords:** geriatric health and nutrition, gut microflora, healthy aging, nano-biotechnology, nanocarriers, probiotics and microbiome, targeted drug delivery

## Abstract

The geriatric population, comprising ages 65 and above, encounters distinct health obstacles because of physiological changes and heightened vulnerability to diseases. New technologies are being investigated to tackle the intricate health requirements of this population. Recent advancements in probiotics and nanotechnology offer promising strategies to enhance geriatric health by improving nutrient absorption, modulating gut microbiota, and delivering targeted therapeutic agents. Probiotics play a crucial role in maintaining gut homeostasis, reducing inflammation, and supporting metabolic functions. However, challenges such as limited viability and efficacy in harsh gastrointestinal conditions hinder their therapeutic potential. Advanced nanotechnology can overcome these constraints by enhancing the efficacy of probiotics through nano-encapsulation, controlled delivery, and improvement of bioavailability. This review explores the synergistic potential of probiotics and advanced nanotechnology in addressing age-related health concerns. It highlights key developments in probiotic formulations, nano-based delivery systems, and their combined impact on gut health, immunity, and neuroprotection. The convergence of probiotics and nanotechnology represents a novel and transformative approach to promoting healthy aging, paving the way for innovative therapeutic interventions.

## Introduction and background

Aging is a lifelong process. As per the World Health Organization (WHO), aging is the accumulation of cellular and molecular damages in a person’s body over time resulting in a gradual decline of physical and mental health [1]. According to the United Nations, the number of people over the age of 65 was 761 million in 2021 worldwide and this number is expected to reach 1.6 billion by 2050 [2]. Aging leads to many disorders and increases the susceptibility of the elderly to many diseases. Conditions like chronic constipation, osteoarthritis, chronic obstructive pulmonary disease, dementia, diabetes, hypertension, depression, hearing loss, cataracts, back and neck pain, frailty, urinary incontinence, delirium, falls, and pressure ulcers are common in the aging population. The individuals can also suffer from more than one disease at a time, leading to geriatric syndromes [1].

Gut microbial colonization commences at birth and attains maturity in adults [3]. Nutritional frailty, referring to a significant decline in weight, muscle mass, strength, or essential physiological reserves due to aging, hinders older adults' ability to meet their nutritional requirements [4]. As individuals cross the age of 70, there are alterations in the digestive and nutrient absorption processes in the gastrointestinal tract, which impact the composition of the gut microbiome. The gut microbiota composition of the elderly undergoes changes influenced by various factors, including age, diet, lifestyle, medications, immunity, geographical location, and co-morbid conditions [5]. Dietary habits play a crucial role in shaping the gut microbiota. Changes in eating patterns, reduced fiber intake, and alterations in nutrient absorption can influence microbial composition [6].

Probiotics are microorganisms that colonize the intestine of the host and show beneficial effects by improving digestion and inhibiting the growth of harmful bacteria. Probiotics are also known to regulate the host’s body systems, like the immune system, nervous system, etc., in a beneficial manner [7]. Probiotics, prebiotics, and dietary interventions are being explored as potential strategies to modulate the gut microbiota in the elderly and promote health. The National Institutes of Health states that the seven primary microorganisms commonly used in probiotic food products are *Lactobacillus*, *Bifidobacterium*, *Saccharomyces*, *Streptococcus*, *Enterococcus*, *Escherichia*, and *Bacillus *[8]. 

Cognitive impairment is a result of aging leading to many neurodegenerative diseases. The plausible involvement of gut microbiome in cognitive impairment has been extensively investigated. The possible correlation between chronic constipation and cognitive decline has also been studied by few researchers. As the gut microflora declines over aging, chronic constipation in the geriatric population is reported almost 20% more as compared to young adults. Understanding the dynamic nature of the gut microbiota in the elderly is crucial for developing interventions that can positively impact health outcomes. Ongoing research continues to uncover the intricate relationships between gut microbiota composition, aging, and health [6, 8].

Nanoscience has revolutionized the pharmaceutical industry by enabling the production of improved therapeutic drugs with enhanced efficacy and lower toxicity. With the help of advanced nanotechnology, controlled-release probiotics can be achieved, preventing overdosing in older adults [9]. Advanced technologies like nanoencapsulation and nanoemulsions are probiotic delivery technologies that can protect probiotic microorganisms from heat, moisture, pH, and digestive tract harshness, improving survival and efficacy. This stabilizes probiotics throughout storage and transport, extending their shelf life. Nanoparticles can also target specific gastrointestinal tract locations to release probiotics when needed. Nanotechnology improves probiotic stability, potency, and administration, boosting their use in functional foods, nutritional supplements, and medicinal treatments.

This review covers the combined impact of probiotics and advanced nanotechnology in improving geriatric health. It provides a comprehensive analysis of how these two advanced approaches can work together to promote healthy aging and improve the quality of life in elderly individuals.

## Review

Research methodology

Search Strategy

A systematic literature review was conducted following the Preferred Reporting Items for Systematic Reviews and Meta-Analyses (PRISMA) guidelines. A comprehensive search was performed across PubMed, Scopus, Web of Science, and Google Scholar for articles published between 2010 and 2025. The search terms included: Probiotics AND geriatric health, Gut microbiota AND aging; Nanotechnology AND probiotics, Nanoencapsulation AND probiotic delivery, Aging AND probiotics. Boolean operators (AND/OR) were used to refine search results, and reference lists of key articles were manually reviewed to identify additional relevant studies.

Study Selection Process

**Figure 1:**
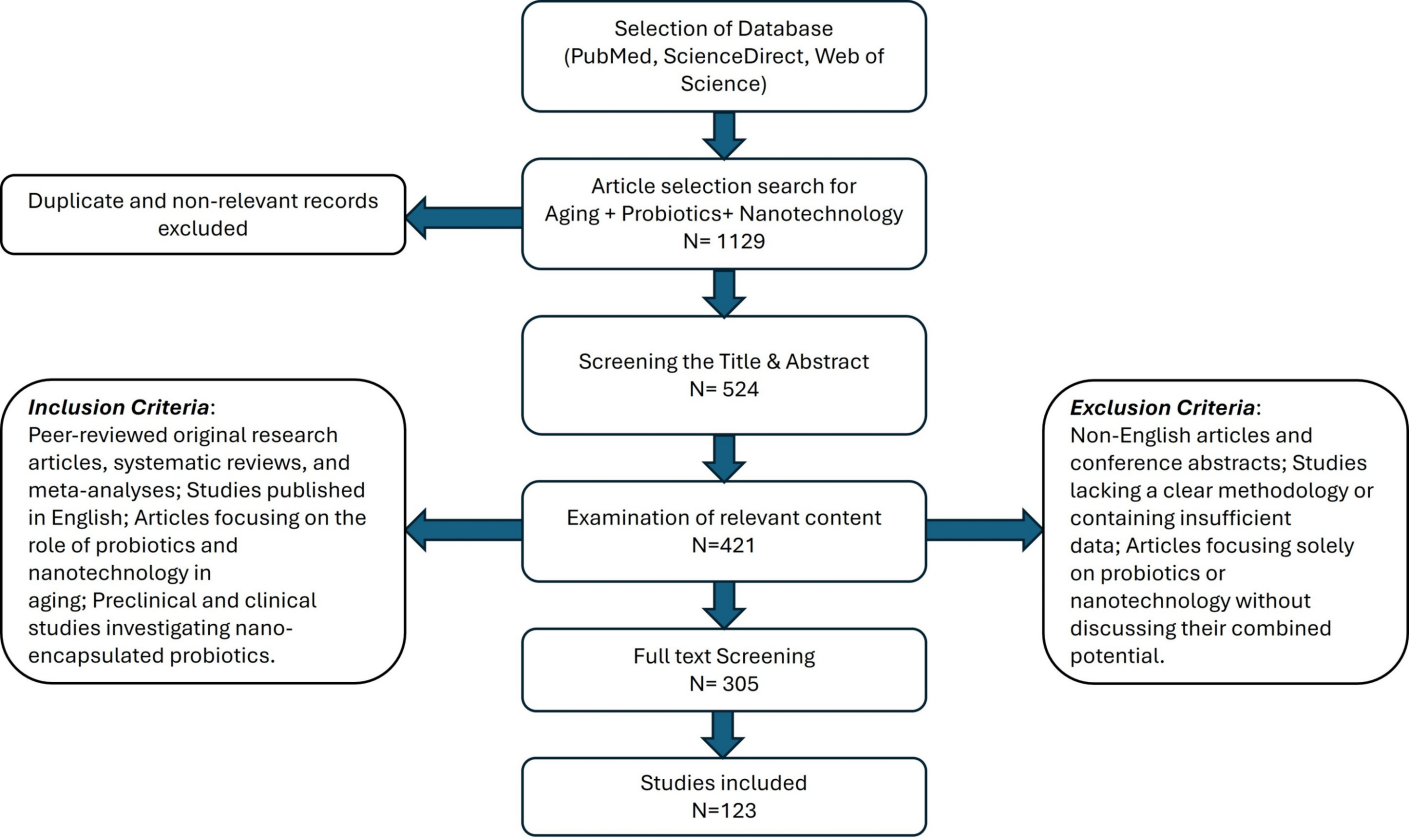
PRISMA flowchart outlining study selection process. The selection process comprised four steps: identification, screening, eligibility, and final inclusion. Identification of studies was primarily through the electronic literature search. Screening involved title screening and removal of duplicate records. Assessing eligibility was based on the inclusion and exclusion criteria and full-text screening. Studies that passed all phases of eligibility screening were included in the review. PRISMA: Preferred Reporting Items for Systematic Reviews and Meta-Analyses

The initial search retrieved 1129 articles. The PRISMA flowchart (Figure 1) has been included to illustrate the selection process. The inclusion and exclusion criteria are given in Figure 1. After removing duplicates, titles and abstracts were screened for relevance (n= 524). A full-text review was conducted on 305 articles, of which 123 met the inclusion criteria. Discrepancies in selection were resolved through discussion among authors. 

The gut microbiota

The initiation of colonization of gut bacteria in the host is a subject of intense discussion. Nevertheless, there are reports indicating that colonization commences during the prenatal stage, while the fetus is still in the uterus [10]. The diversity of bacterial colonies in a newborn is influenced by factors like the method of birth, feeding practices, antibiotic usage, and environmental conditions [11,12]. Babies born through the vaginal canal have abundant populations of beneficial bacteria such as *Lactobacilli*, *Bacteroides*, and *Prevotella* [13,11]. In contrast, babies delivered by cesarean section have higher levels of potentially harmful bacteria including *Clostridium* and *Escherichia* species [14-16]. The composition of microbial communities in newborns is also influenced by the method of feeding. Breast-fed infants harbor *Bifidobacterium*, *Lactobacilli*, *Staphylococci*, and *Streptococci*, whereas formula-fed infants have *Bacteroides*, *Clostridia*, and Proteobacteria [14]. Based on 16srRNA gene research, the primary phyla of bacteria that inhabit the gut are Bacteroidetes and Firmicutes, with Proteobacteria and Actinobacteria following suit [17,18]. The gut microbiota reaches a stable composition once reaching adulthood and thereafter deteriorates as aging progresses [19]. The alteration in the gut microbiota is a condition known as dysbiosis. A decline in viable *Bacteroides* counts with progressive age has been reported by several studies [20]. Some beneficial bacteria, such as *Bifidobacterium* and *Lactobacillus*, may decrease in abundance. These bacteria are involved in the fermentation of dietary fibers and the production of short-chain fatty acids (SCFAs). There might be an increase in opportunistic pathogenic bacteria, which can contribute to inflammation and dysbiosis [21]. This shift may be associated with age-related changes in the immune system. Emerging research underscores the significant impact of gut microbiota dysbiosis on various age-related disorders, summarized in Table 1.

**Table 1:** Impact of gut microbiota dysbiosis on various age-related disorders ↑ denotes an increasing trend and ↓ denotes a decreasing trend.

Age-Related Disorder	Impact of Gut Microbiota Dysbiosis	Altered Microbial Flora	Potential Role of Probiotics	References
Cognitive Decline & Neurodegenerative Diseases (e.g., Alzheimer's, Parkinson's)	Increased gut permeability, neuroinflammation, altered gut-brain axis	↓ *Bifidobacterium*, ↓ *Lactobacillus*, ↑ Proteobacteria, ↑ *Clostridium*	Restores gut-brain communication, reduces neuroinflammation	[22,23]
Cardiovascular Diseases	Elevated trimethylamine-N-oxide (TMAO) levels, systemic inflammation	↑ *Escherichia coli*, ↑ *Desulfovibrio*, ↓ *Bacteroides*	Lowers TMAO, reduces blood pressure, and modulates lipids	[24,25]
Osteoporosis	Reduced calcium absorption, increased inflammation, altered bone metabolism	↓* Bifidobacterium*, ↓* Lactobacillus*, ↑ *Clostridium*	Enhances calcium absorption, modulates bone health	[26,27]
Diabetes and Metabolic Syndrome	Insulin resistance, chronic inflammation, altered short-chain fatty acid (SCFA) production	↑ Firmicutes, ↑ Proteobacteria, ↓ *Akkermansia muciniphila*	Improves insulin sensitivity, restores SCFA balance	[28,29]
Obesity	Increased Firmicutes/Bacteroidetes ratio, low microbial diversity	↑ Firmicutes, ↓ Bacteroidetes, ↓ *Bifidobacterium*	Balances gut microbiota, reduces fat accumulation	[30, 31]
Inflammatory Bowel Disease (IBD) & Gastrointestinal Disorders	Chronic inflammation, disrupted gut barrier function	↓* Faecalibacterium prausnitzii*, ↑ *Enterobacteriaceae*	Strengthens gut lining, reduces inflammation	[32,33]
Sarcopenia (Age-Related Muscle Loss)	Impaired amino acid metabolism, inflammation-driven muscle loss	↓ *Lactobacillus*, ↓ *Bifidobacterium*, ↑ *Clostridium*	Supports protein metabolism, reduces muscle degradation	[34,35]
Immune System Decline (Immunosenescence)	Reduced microbial diversity, increased pro-inflammatory cytokines	↓ *Bifidobacterium*, ↑ *Enterobacteriaceae*, ↑ *Clostridium*	Enhances immune response, regulates inflammation	[18,36]
Depression and Anxiety	Altered gut microbiota composition affecting serotonin production	↓ Bifidobacterium, ↓ Lactobacillus, ↑ Proteobacteria	Supports neurotransmitter balance, improves mood regulation	[37,38]
Chronic Constipation	Impaired gastrointestinal motility and function. Reduced SCFA production.	↓ Bifidobacterium	Balances gut microbiota, restores SCFA balance	[39]

The human microbiome flourishes under ideal growth conditions, which are determined by the body's natural environment. Alterations to the body's natural environment lead to changes in the composition and variety of microbes, as they adjust to the new conditions. This might potentially lead to the development of diseases. Medications, especially antibiotics and drugs affecting the gastrointestinal tract, can have a significant impact on gut microbiota diversity and composition in the elderly. The metabolic functions of the liver and kidneys decline slowly in old age. Due to this, medications can accumulate in the bodies of old persons and may not get metabolized or excreted. This can lead to overdose of medications thereby leading to adverse effects [40]. The decline in *Bifidobacteria* in elderly persons can lead to malnutrition and a low systemic inflammatory status, as *Bifidobacterium* spp. plays a role in stimulating the immune system and metabolic activities.

In addition to changes in the variety of microorganisms, there is also a decrease in metabolites related to microbiota. This decrease may be associated with various aging-related processes such as decreased appetite, weakness, loss of weight, decline in cognitive function, high blood pressure, deficiency in vitamin D, diabetes, arthritis, and loss of muscle mass [28,41,42]. In a systematic review, Tavassol et al. investigated the reported alteration in gut microbiota in relation to obesity and other underlying diseases like diabetes and metabolic syndrome, in older adults [43]. According to the authors, the observational studies done so far are not sufficient to draw a comprehensive conclusion, encouraging more cross-sectional research.

Salazar et al. observed that age plays a significant role in determining the composition of the microbiota among the elderly. They reported elevated levels of *Akkermansia* and *Lactobacillus* in a subset of elderly individuals (>80 years old) compared to the non-elderly adult group (<50 years old) and the younger elderly group (50-80 years old), respectively [44].

Chronic constipation is a prevalent issue among the elderly. Age-related changes in gut microbiota composition can significantly impact gastrointestinal motility and function, contributing to constipation. This shift often results in a reduction of beneficial bacteria such as *Bifidobacterium* and an increase in potentially pathogenic species. Such imbalances can disrupt normal bowel function and are associated with slower intestinal transit times. Beneficial gut bacteria ferment dietary fibers to produce SCFA, which stimulates colonic motility. A decrease in SCFA-producing bacteria may lead to reduced bowel movements. Dysbiosis can compromise the intestinal mucosal barrier and impact bile acid metabolism, leading to low-grade inflammation that affects enteric nervous system function and gut motility. Studies have demonstrated that individuals with chronic constipation exhibit distinct gut microbiota profiles compared to healthy controls. Research indicates that these alterations in microbial composition are associated with constipation symptoms [39].

Probiotics in geriatric health

As per WHO, probiotics are "live microorganisms that confer a health benefit to the host when administered in sufficient doses" [45]. Following are the types of microorganisms used as probiotics: Bacteria of genera *Lactobacillus*, *Enterococcus*, *Escherichia*, *Streptococcus*, *Bacillus*, *Lactococcus*, *Propionibacterium*, and *Bifidobacterium* are most commonly used as probiotics. *Saccharomyces cerevisiae* var. *boulardii* is the only known yeast to be used as a probiotic [46].

Impact of Probiotics on Gut Microbiota Composition in the Elderly

Probiotic bacteria can cause changes in the proportion of populations of different bacterial species in the human gut. They can also affect gene expression patterns of bacteria of other species. In this way, probiotics regulate and stabilize bacterial populations in the human gut. They increase the number of beneficial bacteria and reduce the number of harmful ones [47,48].

Amamoto et al. analyzed the changes in the gut microbiota in a Japanese population and observed the effects of *Lactobacilli *intake on the gut microbiota [46]. Their subjects (age range 66-91 years) were asked to consume fermented milk containing *Lacticaseibacillus paracasei *strain *shirota*, and followed for over a year. They observed changes in the bacterial populations of many families like *Ruminococcaceae, Lactobacillaceae, Prevotellaceae, Bacteroidaceae, Lachnospiraceae*, etc. The probiotic drink given to subjects increased the population of beneficial bacteria and reduced the harmful ones. Further, a study tested the effects of the probiotic bacteria *Lactobacillus rhamnosus *GGATCC53103 on the gut microbiota of 12 individuals aged 65-80 years [47]. The team found that genes related to adhesion, chemotaxis, and flagellar movement of bacteria of genera *Bifidobacterium*, *Roseburia*, and *Eubacterium* were upregulated; thus, indicating an increase in movement and colonization of these beneficial bacterial genera.

A randomized double-blind placebo control trial delved into the effects of *Bifidobacterium longum* BORI and *Bifidobacterium bifidum* BGN4 on the gut microbiota of elderly people [48]. It found that the populations of inflammation-causing bacteria were reduced in the probiotic group compared to the placebo group. These inflammation-causing bacteria were members of the family Prevotellaceae and genera *Eubacterium* and *Allisonella*.

Probiotics and Digestive Health in Geriatric Populations

The trial by Kim et al. also studied digestive habits like the amount and frequency of defecation, passage of gas, stool odor, abdominal distension, the feeling of incomplete evacuation, and intestinal sounds in the geriatric population [48]. They found that passage of gas and abdominal distension were the only digestive habits that showed improvement. The rest of the digestive habits showed no change as compared to the placebo group.

In plant foods, mineral ions like calcium, magnesium, and zinc are bound to a molecule of phytic acid. Due to this, these mineral ions are not absorbed by the human intestinal epithelium. Bacteria from the genera *Bacillus *and *Lactobacillus* produce an enzyme called phytase which degrades this phytic acid thus, freeing calcium, magnesium, and zinc ions in the intestinal lumen. Due to this, the intestinal epithelium can freely absorb these mineral ions. These bacteria also produce SCFA-like propionate and butyrate which create an acidic environment for mineral absorption [7]. A randomized controlled trial (RCT) by Borges et al. found that bacteria like *Streptococcus thermophilus*, *B. longum*, and *Lactobacillus acidophilus* increase potassium absorption in the gut [49].

A meta-analysis by Deng et al. found that the use of probiotics successfully reduced the symptoms of constipation in geriatric patients [50]. Bacteria of genera *Lactobacillus*, *Bacillus*, and *Bifidobacterium* are found to be very useful in reducing the symptoms of constipation.

Probiotics and Immune System Support in Ageing Individuals

The effects of probiotics on the immune system are highly strain-specific. Different strains of bacteria show different effects on the immune system. Moro-Garcia et al. tested the effects of the probiotic organism *Lactobacillus delbrueckii* subsp. *bulgaricus* 8481 on elderly volunteers in a double-blind placebo-controlled study [51]. They found that levels of human beta-defensin 2 were increased, and IL-8 levels were reduced in the probiotic group compared to the placebo group. Finamore et al. tested the effects of a probiotic combination of *B. longum* Bar33 and *Lactobacillus helveticus* Bar13 on the immune function of the elderly [52]. This combination increased the activities of B-lymphocytes, regulatory T-lymphocytes, and natural killer cells and decreased the activity of memory T-lymphocytes compared to the placebo group.

Effect of Probiotics on Other Geriatric Conditions

Kim et al. also studied the effects of probiotics *B. longum* BORI and *B. bifidum* BGN4 on stress, mood, and mental flexibility [48]. They found that probiotics improved mood and mental flexibility and reduced stress in the test group of elderly individuals compared to the placebo group. Serum brain-derived neurotrophic factor (BDNF) levels were higher in the probiotic group than in the placebo group, indicating improved brain functions in the probiotic group. However, these effects were observed after 12 weeks of probiotic treatment.

Zhang et al. performed a systematic review and meta-analysis of studies involving the use of probiotics given during antibiotic treatment of the elderly [53]. They found that the incidence of antibiotic-associated diarrhea (AAD) was reduced in elderly patients when probiotics were administered within 48 hours of antibiotic treatment. Probiotics could not prevent AAD if they were administered after 48 hours. Thus, the effect of probiotics on AAD is closely linked to the time it is given since antibiotic administration.

Borges et al. found that bacterial species like *S. thermophilus*, *B. longum*, and* L. acidophilus* are not effective in chronic kidney disease (CKD) [49]. These bacteria failed to reduce blood levels of urea, uremic toxins, and inflammatory markers in CKD patients.

Potential Risks, Contraindications, and Adverse Effects of Probiotic Supplementation in Geriatric Individuals

While probiotics have demonstrated numerous health benefits in older adults, their use is not without potential risks. Weakened immunity, underlying health conditions, and polypharmacy may influence the safety and efficacy of probiotic supplementation. Elderly individuals may be at an increased risk of infections caused by probiotic bacteria. Cases of bacteremia and fungemia have been reported, particularly with *Lactobacillus* and *Saccharomyces boulardii *strains in critically ill patients [54,55]. In some cases, excessive probiotic supplementation may contribute to small intestinal bacterial overgrowth (SIBO), leading to symptoms such as bloating, abdominal discomfort, flatulence, and altered bowel movements as well as brain fog [56]. Probiotics may also interact with certain medications commonly prescribed to older adults, including immunosuppressants, antibiotics, and antifungal medications [54].

While probiotics offer potential benefits for aging individuals, their use should be approached with caution in certain populations. Careful strain selection, awareness of contraindications, and professional guidance can help maximize the therapeutic effects of probiotics while minimizing potential risks in geriatric healthcare.

Synergies between probiotics and nanotechnology

Nanotechnology in Healthcare

Current medical treatment strategies involve the use of medications to treat different geriatric diseases. However, at high doses, these medications may show adverse effects and may target other organs that are not affected by the disease. At low doses, the medication may be ineffective and will be easily metabolized by the liver and excreted by the kidneys. Here, nanotechnology helps to solve the problem. Nanoparticles containing the right amount of drug and with ligands on the surface that bind the target cells help to guide the drug to the correct target tissue and release the drug gradually. There are many examples of the use of nanoparticles in the healthcare of geriatric patients. For example, nanoparticles with lactoferrin on their surface bind to lactoferrin receptors in the blood-brain barrier (BBB) to cross the BBB. These nanoparticles are helpful in delivering drugs to the brain in cases of Alzheimer’s disease, Parkinson’s disease, Huntington’s disease, etc. In rheumatoid arthritis, nanoparticles can be designed to deliver siRNA to macrophages, thereby changing them from pro-inflammatory to anti-inflammatory and reducing the inflammation of joints. In inflammatory bowel disease, nanoparticles targeting inflammatory immune cells in the gut are loaded with siRNA or anti-inflammatory drugs. These nanoparticles are effective in reducing inflammation in the gut [55].

Nanotechnology Applications in Diagnostics for Age-Related Diseases

Nanotechnology also has applications in the diagnosis of many different diseases. Advances in nanotechnology have helped to create more sensitive diagnostic tests. Nanoparticles are functionalized with antibodies specific to a biomarker of a disease. These nanoparticles are then used to detect the biomarkers in blood, urine, saliva, etc. The nanoparticles bind to the biomarkers of interest and form agglomerates that have different optical, magnetic, or fluorescent properties. These agglomerates are detected via methods like spectrophotometry, flow cytometry, surface plasmon resonance, etc. These nanoparticles are used to detect biomarkers of cardiovascular diseases, cancer, etc. [56]. All aspects of combining probiotics with nanotechnology for geriatric health are shown in Figure 1.

**Figure 2:**
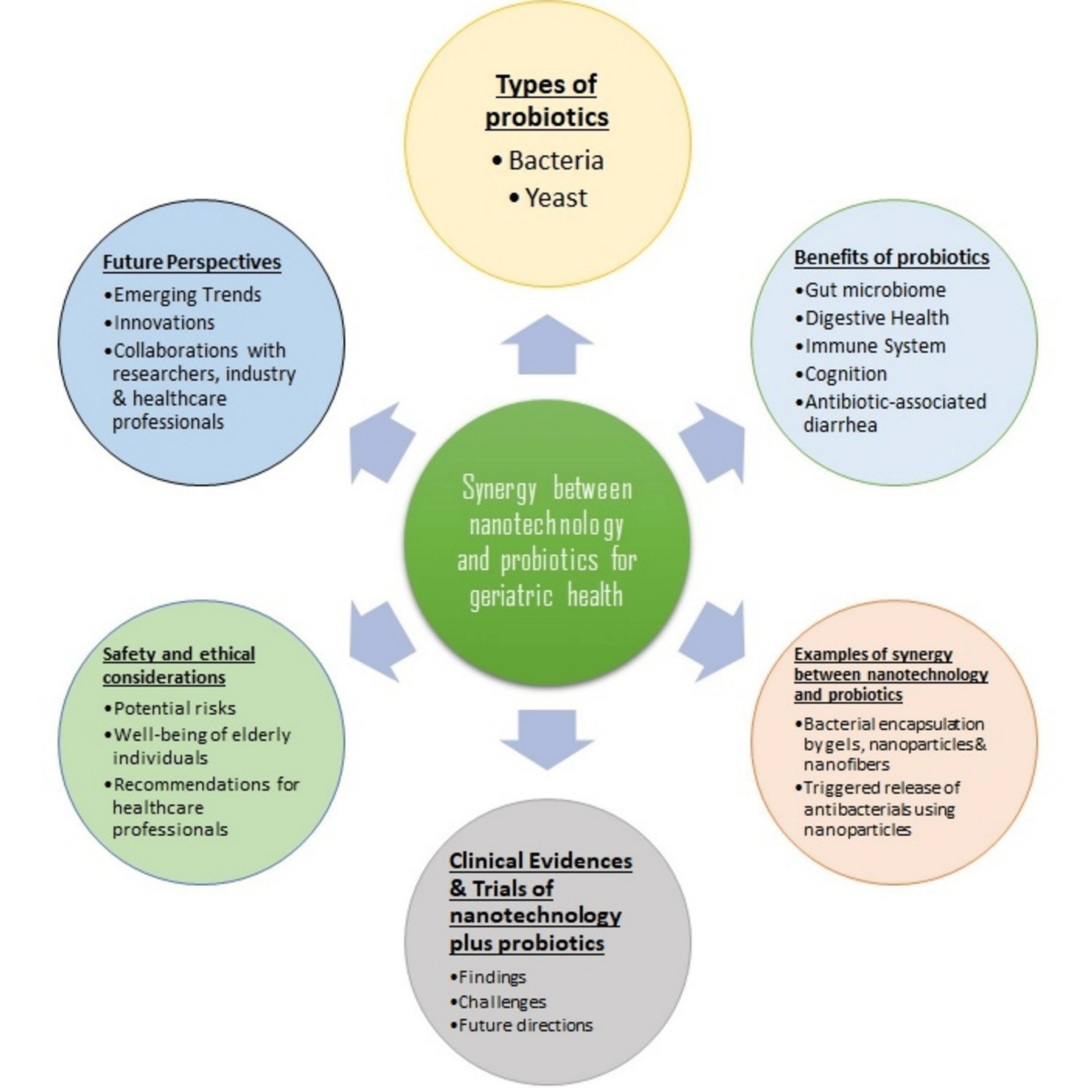
Aspects of combining probiotics in geriatric health by nanotechnology Image Credits: Onkar Kadam

Nanoparticles are also used to locate tumors. Nanoparticles designed to bind on surface biomarkers of tumors are used. These nanoparticles bind on the tumor surface and fluoresce or show different magnetic properties. Then the tumor is visualized and located using methods like MRI and PET scan [56].

Applications of Nanotechnology to Deliver Probiotics in the Human Gut

Many probiotic organisms are prone to being destroyed in unfavorable conditions. For example, *Lactobacillus rhamnosus* and *B. longum* get injured by the acidity of the stomach and the presence of bile salts in the gut. Anaerobic probiotics like *L. acidophilus *and *Bifidobacteria* do not survive in oxygen-rich environments. Also, probiotic microbes can get destroyed due to improper handling and storage. Thus, it is necessary to encapsulate these microbes in a suitable material that is resistant to these unfavorable conditions. However, at the same time, the encapsulating material should degrade in the intestine so that it releases the probiotic microbes in the intestinal lumen for colonization. Nanotechnology has the potential to create such materials with these unique properties [57]. Nanotechnology increases intestinal epithelial absorption of probiotics, improving bioavailability. Nanoparticles help probiotic bacteria pass the intestinal barrier, improving their medicinal effects. Nanotechnology allows probiotic-bioactive medication combinations. Encapsulating probiotics and other medicinal compounds in nanoparticles have synergistic effects that promote health. Nanotechnology-based biosensors can detect and quantify probiotic bacteria quickly. These biosensors can measure probiotic levels in food, supplements, and biological samples for quality control and efficacy. Nanotechnology can customize probiotic compositions for unique customer needs or health situations. By changing nanoparticle physicochemical properties, probiotic release kinetics and stability can be adjusted.

As mentioned above, bacteria of the genus *Bifidobacterium* do not do well in the human gut as well as in oxygen-rich environments [57]. Hence, Fayed et al. synthesized microcapsules made up of gum Arabic and alginate [58]. These capsules contained bacteria of the genus *Bifidobacterium* and inulin-containing poly (lactic-co-glycolic acid) (PLGA) nanoparticles. The nanoparticles acted as a food source for bacteria during storage of capsules. These capsules were exposed to solutions that mimicked stomach acid and intestinal bile and increased the viability of bacteria in these solutions compared to free bacteria. Also, a slow release of inulin and bacteria was observed in these solutions. Fayed et al. also tested these same capsules in yogurt [59]. The capsules were able to keep the bacteria viable and were able to release them slowly in yogurt [59].

Encapsulating probiotics in nanoparticles protects them from the harsh gastrointestinal environment and external variables like air, temperature, and light during storage and handling, increasing their survival and efficacy. It also allows probiotics to be added at various quantities and precise and regulated release of encapsulated components in the digestive tract. These benefits demonstrate the low-to-high potential of encapsulation. Encapsulation can improve probiotic product performance and efficacy, as shown by these benefits. In humans, nanoparticle-encapsulated drugs have not been standardized. Research in this field is scarce, giving researchers many opportunities to produce functional food products. Researchers can create products with multiple functions and improved active ingredient delivery in the gut by co-encapsulating bioactive compounds and probiotics. This presents a promising avenue for developing innovative food formulations with improved health benefits and targeted effects on the body [58,59]. 

Table 2 summarizes various applications of nanotechnology for probiotic delivery.

**Table 2:** Examples of applications of nanotechnology for probiotic delivery SGC: simulated gastrointestinal conditions; NP: nanoparticles; ROS: reactive oxygen species; DSS: dextran sulfate sodium

Bacterial Strain	Material to Encapsulate Bacteria	Nanoparticles Used and Their Purpose	Conditions Tested on and Their Results	Reference No.
*Pediococcus pentosaceus* Li05	Alginate and gelatin	Magnesium oxide (MgO) NPs used to fill the pores in alginate-gelatin microcapsules. MgO NP neutralized acidity in and around the microcapsule	Probiotics in NP-free microcapsules tolerated more heat than NP-loaded microcapsules. Probiotics in NP-loaded microcapsules tolerated SGC better than NP-free microcapsules	[60]
*Lactobacillus rhamnosus* KLDS 1.0320, *Lactobacillus* *acidophilus* KLDS 1.0327, *Lactobacillus* *plantarum* KLDS 1.0328, *Lactobacillus* *casei* KLDS 1.0338	Pullulan-gum Arabic nanofibers were used. Encapsulation done by electrospinning and freeze-drying	No nanoparticles used	Encapsulated probiotics were stored at 4⁰C for 28 days. Probiotics encapsulated by electrospinning showed more viability than those encapsulated by freeze-drying after the storage period.	[61]
*Bacillus coagulans*, *Escherichia coli*	Bionanocomposites made of bacterial cellulose, pectin, and extract of fungus Schizophyllum commune	No nanoparticles used	Encapsulated probiotics were tested under two different conditions, SGC and microwave drying, and remained more viable than free cells. It was found that bacterial cellulose was a better prebiotic for *B. coagulans* than to *E. coli.*	[62]
L. acidophilus, L. rhamnosus	Three layered encapsulations with innermost calcium alginate, middle chitosan, and outermost Eudragit S100 NPs	Eudragit S100 NPs were used as the outermost covering of probiotic capsules. Along with chitosan, these NP-covered pores are in the alginate layer.	Tested on SGC. Encapsulated bacteria were more viable than free cells. Alginate capsules with both chitosan and NP layers showed more bacterial viability than alginate capsules with only chitosan layers.	[63]
Pediococcus acidilactici	Three-layered encapsulations with innermost alginate, middle chitosan, and outermost alginate.	Phthalyl inulin NP used. These NPs triggered the release of antibacterial peptide pediocin from *P. acidilactici*.	These capsules were added to a bacterial culture of *Salmonella gallinarum*. The release of pediocin by NP-containing *P. acidilactici* capsules was more than capsules without NP but containing *P. acidilactici*.	[64]
Lactobacillus plantarum	No encapsulation of bacteria and nanoparticles was done.	Phthalyl pullulan nanoparticles were used to trigger more plantaricin production than normal in *L. plantarum.*	Phthalyl pullulan NP-internalized *L. plantarum* was cocultured with *E. coli* K99 and *Listeria monocytogenes *and showed antibacterial activity against both of them. However, the authors of this study did not test it under SGC.	[65]
*Escherichia coli *Nissle 1917	Encapsulation of bacteria was done by polynorepinephrine	Hyaluronic acid-poly (propylene sulfide) nanoparticles were attached to the surface of the polynorepinephrine capsule. These NP acted as scavengers for ROS	Tests were done on DSS-induced colitis mouse models. The encapsulated bacteria with NP on surface showed prophylactic and therapeutic efficacy compared to untreated mice. The NP cleared ROS in DSS mice. Encapsulated bacteria promoted the growth of healthy bacteria in the gut of DSS mice. This study has potential to help geriatric patients with inflammatory bowel disease (IBD).	[66]
*L. casei* Zhang	No encapsulation was done	Inorganic nanophosphors were synthesized and coated with deoxyribonucleic acid (DNA). These DNA-coated nanophosphors were absorbed by *L. casei* Zhang.	These bacteria with nanophosphors were introduced in mice. Imaging studies of these mice showed luminescence at different body parts which indicated the location of bacteria in these body parts	[67]
*E. coli *Nissle 1917	Encapsulation was done by alginate followed by a layer of chitosan NPs	Chitosan NPs formed the outer covering of the alginate capsules.	Encapsulated bacteria showed improved viability in SGC than free cells and inhibited the growth of pathogenic bacteria, *Campylobacter jejuni*.	[68]
*L. acidophilus *LA5, *L. rhamnosus* 23527 LGG, *Bifidobacterium bifidum*, *Bifidobacterium* *animalis*	Nanofibers made of sodium alginate and corn starch	No NPs were used	Tested on SGC. Encapsulated bacteria showed improved viability than free cells.	[69]
*L. acidophilus* ATCC 4356, *Lactobacillus* *delbrueckii* ssp. *bulgaricus* ATCC 11842, *L. casei* ATCC 393, *Lactobacillus* *gasseri *ATCC 33323, *Lactobacillus* *paracasei* ATCC 25302, *L. plantarum* ATCC 8014, *Lactobacillus* *reuteri* ATCC 55730, *L. rhamnosus *ATCC 53103, *Lactobacillus* *salivarius* ATCC 11741, *Lactococcus lactis* ssp. *cremoris* MG1363	Electrospun nanofibers made up of poly (ethylene oxide)	No NPs were used	All strains of bacteria used in this study were viable after electrospinning	[70]
Enterococcus faecium	Nano-emulsion made up of whey protein concentrate, gum Arabic, inulin, and coconut oil. Inulin acted as prebiotic. The bacteria was entrapped in the globules of this emulsion	No NPs were used	Storage at 27⁰C and 4⁰C. Bacteria remained viable after storage.	[71]
L. plantarum	Macrogel made up of cellulose fibers and nanofibers	No NPs used	Tested in SGC. The bacteria remained viable in SGC, and the gel showed gradual release of bacteria in SGC.	[72]
L. plantarum	Encapsulation of bacteria by cellulose microgels followed by a coating of calcium alginate	No NPs used	Tested in SGC. The bacteria remained viable in SGC, and the gel showed gradual release of bacteria in SGC.	[73]
*L. acidophilus* (DMS 20079),* Lactobacillus johnsonii *(DMS 10533), *L. casei* (DSM 20011), *B. longum *subsp. *infantis* (*B. infantis*) (DMS20088), *Saccharomyces cerevisiae* var. *boulardii*	Cellulose sulphate microspheres	No NPs used	Encapsulated bacteria survived in SGC. When encapsulated bacteria were fed to mice, the capsules released bacteria in intestine and the bacteria colonized in the intestine	[74]
*B. bifidum* TISTR2129, *Bifidobacterium breve *TISTR2130, and *L. acidophilus* TISTR1338	Wall material made by combining jackfruit inner skin fiber, soyabean oil, and whey protein isolate	No NPs used	Tested under SGC and freeze-drying conditions. The wall material successfully protected the bacteria encapsulated within it under both conditions.	[75]
Kluyveromyces lactis	Nanocomposite hydrogels made of gelatin and graphene oxide	No NPs used	Encapsulated bacteria showed moderate viability under SGC.	[76]

A novel and underutilized strategy to address this issue is to combine nanotechnology with food technology. Nanotechnology provides various opportunities for the development and analysis of nanomaterials or nanoparticles with sizes ranging from 1 to 100 nm. These nanoparticles can be used to encapsulate probiotics, bioactive components released by probiotic microorganisms, prebiotics, or symbiotics, resembling capsules. Consequently, nano-structured probiotics, prebiotics, and symbiotics were shielded from harmful gastrointestinal conditions, guaranteeing their secure delivery to the intended location [77]. The distinct functional properties of these molecules can be influenced by the physical, chemical, biological, and mechanical qualities of the nanomaterial employed [57]. Nanomaterials can be derived from natural sources such as starch, cellulose, protein, and lipids, or they can be made synthetically using metal-based polymers. An optimal nanomaterial must possess biocompatibility, biodegradability, and bio tolerance, and be widely acknowledged as safe (GRAS) for utilization in probiotics, prebiotics, and symbiotics [78].

The microbiome signature, next-generation probiotics, metabolome, microbial consortia, and nano-nutraceuticals are advanced technologies that have the ability to address nutritional, scientific, and health-related challenges. The continuous progress in microbiome-related knowledge has created new opportunities for research on probiotics and prebiotics. The advancement of these therapies related to the microbiome, along with the shifting acceptance by various stakeholders (consumers, researchers, policymakers, industries, and regulatory agencies), signifies a time of significant transformation (from traditional fermented foods to more advanced microbiome-related science). Therefore, nano probiotics and nano prebiotics offer a cost-efficient and encouraging method to provide health advantages to an individual at the nanoscale. They ensure safety and effectiveness when utilized to manage health conditions, especially for patients who are not suitable for traditional pharmacological treatment [79].

Regulatory, Ethical, and Safety Challenges of Nanoparticle-Enhanced Probiotics

Despite various advantages, the safety and regulatory concerns of nanostructured probiotics and prebiotics still need to be researched and addressed. The scientific community in the fields of food technology, biotechnology, and nanotechnology is actively working to discover more effective methods for preventing and treating diseases [79]. The integration of nanoparticles into probiotic formulations introduces several regulatory, ethical, and safety considerations.

Nanoparticles, due to their diminutive size and high surface area-to-volume ratio, can exhibit unique interactions with biological tissues. Potential toxicity concerns include unintended effects from their ability to cross biological barriers, such as the BBB, and the long-term effects of nanoparticle accumulation in the body. These factors necessitate rigorous safety assessments, including comprehensive toxicological studies, to evaluate the biocompatibility and potential adverse effects of nanoparticle-enhanced probiotics. Ensuring that these products do not elicit harmful immune responses or disrupt normal physiological processes is paramount for their safe integration into consumer health regimens. Regulatory bodies, such as the United States Food and Drug Administration (FDA), are actively developing guidelines tailored to nanotechnology-based products, including those involving probiotics. Naturally derived nanomaterials are gaining more attention compared to chemically synthesized materials, as the end product can deliver more sustainability, lower toxicity, improved functionality, and increased bioactivity [80,81].

The application of nanotechnology in probiotics raises ethical questions related to consumer autonomy and informed consent. The novelty and complexity of nanomaterials may lead to public apprehension, particularly if consumers are not adequately informed about the presence and purpose of nanoparticles in products. Transparent labeling and public education are essential to empower consumers to make informed choices. Additionally, ethical deliberations must consider the long-term societal and environmental impacts of widespread nanoparticle use, ensuring that technological advancements do not compromise public trust or ecological balance [82].

While the fusion of nanotechnology and probiotics holds promising therapeutic potential, it is imperative to address the accompanying regulatory, ethical, and safety challenges.

Clinical evidence and trials

Clinical trials conducted thus far have examined the alterations in the gut microbiota, immunomodulation, and the influence of probiotics on digestive health, overall well-being, cognitive function, as well as lipids and other biomarkers. Nevertheless, multiple systematic reviews emphasize the necessity for additional randomized research to determine the genuine advantageous impacts of probiotics on cognitive function, mood, metabolic indicators, and age-related frailty. 

Overview of Studies Combining Probiotics and Nanotechnology in Geriatric Health

Encapsulating probiotics in nanoparticles protects them from the harsh gastrointestinal environment and external variables like air, temperature, and light during storage and handling, increasing their survival and efficacy. It also allows probiotics to be added at various quantities and precise and regulated release of encapsulated components in the digestive tract. These benefits demonstrate encapsulation's low-to-high potential. Encapsulation can improve probiotic product performance and efficacy, as shown by these benefits. In humans, nanoparticle-encapsulated drugs have not been standardized. Research in this field is scarce, giving researchers many opportunities to produce functional food products. Researchers can create products with multiple functions and improved active ingredient delivery in the gut by co-encapsulating bioactive compounds and probiotics. This presents a promising avenue for developing innovative food formulations with improved health benefits and targeted effects on the body [83].

Table 3 highlights the significant advancements in preclinical research and theoretical frameworks that support the use of nanoparticle-based encapsulation to enhance probiotic functionality. There is a growing interest in nanoencapsulated probiotics for geriatric health, focusing on improving stability, targeted delivery, and therapeutic efficacy. A major area of interest is the potential for nanoencapsulated probiotics in precision medicine.

**Table 3:** Summary of preclinical studies and theoretical frameworks on nano-encapsulated probiotics for geriatric health

Author, Year	Focus	Findings
Panghalet al., 2019 [84]	Microencapsulation techniques for probiotic delivery	The study reviewed various microencapsulation methods aimed at protecting probiotic bacteria during gastrointestinal transit, thereby enhancing their viability and therapeutic efficacy.
Lavanya et al., 2024 [85]	Encapsulation techniques for probiotics in neurodegenerative diseases	Explores advanced formulation techniques, such as microencapsulation and nanoencapsulation, to enhance the viability and therapeutic potential of probiotics in managing neurodegenerative diseases, suggesting potential applications in elderly care.
Butler et al., 2020 [86]	Probiotics reducing antibiotic use in elderly care home residents	The trial found that daily probiotic use was associated with a modest reduction in antibiotic administration for common infections among care home residents, suggesting a role for probiotics in infection management in elderly populations.
Sun et al., 2023 [87]	Biomaterials and techniques for probiotic encapsulation	The study explored various biomaterials and encapsulation methods designed to protect probiotics from adverse environmental conditions, thereby improving their viability and therapeutic potential.
Krsek and Baticic, 2024 [88]	Nanotechnology in neurodegenerative diseases	The study explores nanotechnology-based therapies targeting neurodegenerative disorders prevalent in the elderly, such as Alzheimer's and Parkinson's diseases, indicating promising advancements in treatment strategies.
Machado et al., 2020 [89]	Integration of nanotechnology and probiotics for gut health	Theoretical exploration of combining nanotechnology with probiotics to enhance gut health, discussing potential applications and benefits of this innovative approach.
Giannouli and Karalis, 2022 [90]	Nanotechnology in anti-aging therapies	The study highlights the potential of nanotechnology-based formulations in delivering anti-aging substances, suggesting that nano-delivery systems can enhance the efficacy of these compounds in elderly populations.
Hu et al., 2024 [91]	Nanoparticle-encapsulated probiotics in Alzheimer's disease	Layer-by-layer encapsulation of Lactiplantibacillus plantarum enhanced its viability and bioactivity, leading to improved cognitive function and reduced neurotoxicity in Alzheimer's disease mouse models.
Hutchinson et al., 2021 [92]	Effect of Probiotics on health and immunity	Discusses the efficacy of Probiotics in modifying gut microbiota composition in healthy older adults. Shown moderate effects on immune function. However, the effect of probiotic supplementation on other health outcomes remains inconclusive, highlighting the need for more well-designed studies.
Rees and Moghimi, 2012 [93]	Nanotechnology applications in aging	The article discusses how nanotechnology has begun to enhance the quality of life for the elderly through medical diagnostics, drug delivery, and tissue regeneration, addressing age-related health issues.
Ma et al., 2024 [94]	Nanotechnology in healthcare and associated risks	The review explores the applications of nanotechnology in healthcare, including potential benefits for the elderly, while also addressing safety and environmental concerns associated with its widespread use.
Garcia-Brand et al., 2024 [95]	Stimuli-responsive encapsulation for targeted probiotic delivery	This review focused on the development of stimuli-responsive encapsulation systems that release probiotics in response to specific environmental triggers, enhancing targeted delivery and efficacy.
Banerjee et al., 2024 [96]	Combined use of probiotics and nanovaccines in colorectal cancer prevention	Preclinical studies using animal models indicated that probiotics, when combined with cancer nanovaccines, effectively reduced tumor development in the colon. This suggests a synergistic effect, enhancing the efficacy of cancer prevention strategies.
Koh et al., 2022 [97]	Techniques and materials for probiotic encapsulation	The study reviewed various encapsulation techniques and coating materials aimed at improving the stability and delivery efficiency of probiotics, particularly in non-dairy-based products. Encapsulation was found to significantly enhance probiotic survivability in challenging environments.
Sánchez Y Sánchez de la Barquera et al., 2022 [98]	Probiotics and prebiotics in elderly gut health	The research underscores the association between gut microbiota and frailty syndrome in older adults, suggesting that probiotics and prebiotics may help mitigate age-related diseases by improving gut health.
Razavi et al., 2021 [57]	Nanomaterial-based encapsulation for controlled probiotic delivery	The study examined the use of nanomaterials for encapsulating probiotics, aiming to protect them from harsh gastrointestinal conditions and ensure controlled release at the target site.
Kumarasamy et al., 2024 [99]	Nanoparticles in clinical therapies	This review discusses the diverse applications of nanoparticles in various medical fields, including their potential in enhancing drug delivery and therapeutic outcomes, which could be beneficial for age-related conditions.
Pandey et al., 2025 [100]	Nanoencapsulation of probiotics for gut-brain axis modulation	Nanoencapsulation techniques improved the survivability of probiotics under various processing and storage conditions. In vivo studies demonstrated enhanced gut microbiota composition in rats, suggesting potential benefits for gut-brain axis modulation.
Coutts et al., 2020 [101]	Probiotics, prebiotics, and synbiotics in elderly functional outcomes	The review suggests potential benefits of probiotics, prebiotics, and synbiotics on functional outcomes in older adults, but emphasizes the necessity for more robust clinical trials to confirm efficacy.
Wang et al., 2025 [102]	Encapsulated probiotics for neurological health	Discusses the potential of biologically macromolecule-encapsulated probiotics in preventing and treating neurological disorders, emphasizing the need for clinical trials to validate efficacy, particularly in aging populations.
Besora-Moreno et al., 2024 [103]	Probiotics in elderly health	Probiotic supplementation may counteract age-related shifts in gut microbiota, potentially promoting healthy aging. However, randomized controlled trials have shown conflicting results, indicating the need for further research.
Chen et al., 2025 [104]	Nanoparticle-enhanced probiotics for disease treatment	Reviews the innovative application of nanoparticle-armored engineered probiotics for precise disease treatment, addressing physiological barriers associated with oral administration, with potential implications for geriatric health.

A key area of research is neurological health, where researchers have explored how nanoencapsulation can enhance probiotic effects in neurodegenerative diseases, particularly Alzheimer’s disease [91,102]. The ability of encapsulated probiotics to modulate the gut-brain axis suggests promising therapeutic implications for aging populations. Additionally, stimuli-responsive and controlled-release probiotics were investigated, ensuring precision in probiotic administration [95,57]. Meanwhile, the combination of probiotics with nanovaccines suggests potential applications in colorectal cancer prevention [96]. Additionally, concerns regarding the potential toxicity of nanomaterials, ethical considerations, and regulatory approvals must be addressed before widespread application. While Emon et al. explored the potential benefits of microencapsulation for probiotic delivery [82], the long-term impact of nanomaterial interactions with the human microbiome and systemic health remains unclear.

Chen et al. discussed the theoretical applications of nanoparticle-armored probiotics in treating specific diseases, highlighting how nanoengineering can enable targeted probiotic release at disease sites [104]. Many studies emphasized the role of biomaterials in protecting probiotics from adverse environmental conditions, enhancing their survivability and effectiveness [87,97].

Despite these promising developments, the translation of nano-probiotic technologies from preclinical models to clinical applications remains limited. While most of the researchers provided valuable insights into the integration of nanotechnology with probiotics, their findings primarily remain within theoretical frameworks or in vitro models. Further clinical trials are needed to establish the safety, efficacy, and regulatory compliance of these formulations, particularly for use in geriatric populations.

Safety and ethical considerations

Potential Risks Associated With Combining Probiotics and Nanotechnology

In recent years, nanotechnology has drawn a lot of attention for use in biomedical applications [105]. The key factors influencing the toxicological effects of nanoparticles include their size, shape, surface charge, and ability to pass biological membranes [106]. Nanoparticles have a greater absorption rate, tiny particles that can accumulate and cause both direct and indirect toxicity to cells and tissues in organs [105]. In complicated biological systems, nanoparticles have the potential to produce pro-oxidant species, which might lead to uncontrollably high levels of reactive oxygen species (ROS), reactive nitrogen species (RNS), and free radicals. This would promote oxidative stress, inflammatory conditions, and other associated pathological states, such as immunological toxicity. The inherent toxicity of polymers and leftover solvents may cause immunological responses that endanger the environment and public health [106]. The consumption of foods containing bioactive chemicals loaded with nanoparticles has been linked to the effects on the liver, kidney, and spleen, and perhaps allergic responses. Additionally, polyethylene glycol (PEG) -grafted liposome infusions have been linked to non-IgE-mediated symptoms of hypersensitivity. More research has shown that exposure to nanoparticles, such as carbon black, silicates, titanium dioxide, and iron oxide, can cause oxidative damage and gastrointestinal tract inflammation [107]. Patients with gastrointestinal disorders typically use medications coated with chitosan-silver nanoparticles (ChAgNPs) (silver or titanium dioxide), which are sold in the market. However, these specific medications have the effect of destroying the probiotic gut microbial community, which causes the immune system to deteriorate over time [108].

Ethical Considerations in Geriatric Research and Interventions

Limited resources and an aging population that is growing rapidly have presented significant challenges for health policymakers. This requirement has incentivized researchers to concentrate their efforts on geriatric research, with the aim of investigating the natural history of chronic illness, identifying markers of health status, and formulating plans for the efficient use of limited healthcare resources [109]. Many researchers who work with older adults have not taken advantage of current advancements in research, despite the fact that there is an abundance of research activities. The advancement of science and medicine for elderly individuals is now at a standstill [110]. When developing their investigative techniques, research methodologists frequently overlook investigative vulnerabilities specific to geriatric research. A research question's perspective and underlying values should be clearly examined in any geriatric study by examining health service delivery, quality of life, and access to medical treatment. The fundamental moral dilemma in human research stems from the fact that, on one hand, the study initiative, aims to generate broadly applicable new knowledge, and is anticipated to advance medical science in a way that will improve people's health; on the other hand, a human being is being used to generate benefits that might not benefit them, putting them at risk and raising the possibility that they will be coerced into doing research [111]. 

There are four broad ethical concerns in human research that are particularly relevant to studies that deal with older people: (i) Objectives and intentions of the study: this ethical dilemma involves deliberations over the research's use, need, and overall priority relative to other studies that may be conducted, in addition to the motive of the sponsor or investigator, (ii) Analysis of risks and benefits: a study shouldn't be carried out until the expected advantages to individuals, groups, or society justify the risks or damages (social, psychological, or physical) to the subject, other people, or society as a whole, (iii) Choice of subjects: this asks if a certain group of study participants is being utilized disproportionately, and (4) Conscientious agreement: a crucial condition for conducting human research is that the subject of the study agrees to engage voluntarily in the activity or endeavor. Apart from these ethical issues, there are more issues that are related to geriatric research. for example, the following are addressed in research on anti-aging: health issues that make older people more susceptible to being chosen for study, conditions that make it difficult for older people to give their free and informed consent, surrogate consent and guardianship issues, and unique issues with competence and free consent for older people living in institutions [112].

Ensuring the Well-Being of Elderly Individuals in Experimental Settings

Growing older is a natural and ongoing process, but if handled well, it may be a chance for people to live longer and in good health [113]. Physiological changes that occur in the gastric phase of aged people are increased average pH and reduced levels of enzymes (e.g. pepsin, lipase), and the impact this causes is decreased effectiveness of digestion, altered absorption, and altered bioavailability of dietary ingredients [114-116]. On the other hand, in the intestinal phase, there is reduced emptying of the stomach, altered composition of bile and pancreatic secretions, decreased levels of certain enzymes (such as pancreatin, lipase, and α-amylase), modified reactions to hormones (such as ghrelin and cholecystokinin), and altered intestinal motility and microbiota. This results in longer transport times which decreases the effectiveness of digestion, altered absorption, and bioavailability of dietary components impaired nutrition absorption, a rise in gastrointestinal disorders and infections, less appetite and thirst, and heightened sensitivity to the satiating effects [117-119]. The various effects of probiotics on geriatric health are shown in Figure 2.

**Figure 3:**
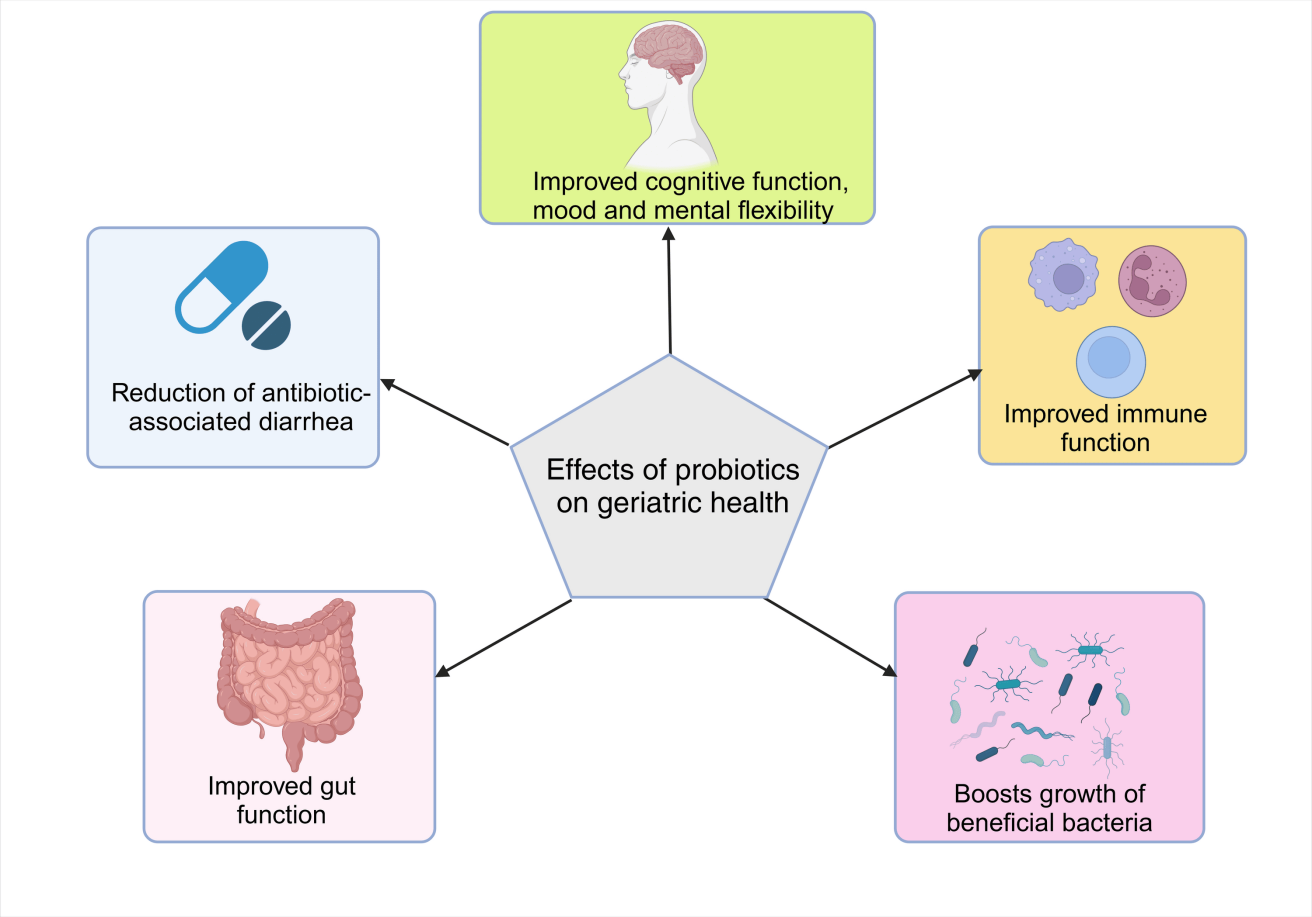
Various effects of probiotics on geriatric health Image Credit: Authors

Elderly individuals often experience changes in gut microbiota due to aging, which can affect digestion, immunity, and overall health. Maintaining a balanced gut microbiome is crucial for their well-being. In an interesting experiment, Catto et al. utilized an in-vitro gut model to simulate human intestinal conditions [119]. The model was exposed to dietary silver nanoparticles (AgNPs) (1 μg/mL) to assess their impact on the composition and function of gut microbiota. Additionally, the study explored whether administering probiotics (*Bacillus subtilis*) could mitigate any adverse effects induced by AgNPs. Probiotics helped stabilize key metabolic pathways, demonstrating a protective effect against AgNP-induced gut microbiota changes. The findings from this study suggest that while exposure to AgNPs might disrupt gut microbiota, the use of probiotics could serve as a protective strategy to preserve gut health. This insight is valuable for developing dietary recommendations and interventions aimed at enhancing the health and quality of life of the elderly population.

Recommendations for Healthcare Professionals in Incorporating Probiotics and Nanotechnology into Geriatric care

The food and pharmaceutical sectors have made extensive use of probiotic microencapsulation. Probiotics are protected using microencapsulation, which involves placing the microorganisms within tiny capsules [57]. Probiotic microencapsulation has several benefits for the medical field. Increasing the longevity of probiotic bacteria by shielding them from the severe conditions of the gastrointestinal tract and unfavorable interactions with outside elements (such as oxygen, temperature, and light during handling and storage). Guaranteeing that the encapsulated components are released into the gastrointestinal system in a controlled and targeted manner at the appropriate concentrations; encouraging the probiotics' efficient adherence to the tissues of the gut mucosa; and enhancing the capacity to provide probiotics in the appropriate amounts (varying from low to high levels). The potential for using synbiotic multiparticulate microcapsules, which include probiotic bacteria and PLGA nanoparticles loaded with inulin, to improve the encapsulated probiotics for four weeks and the survival rate is above that of probiotics added to yogurt freely [59]. Probiotics with induced nanoparticles show antimicrobial activity in one of the studies conducted by Cattò et al. [119]. 

Probiotics with induced nanoparticles show antimicrobial activity in one of the studies by Kheradmand et al. [120]. Investigations have shown how selenium dioxide affects* L. plantarum* and *L. johnsonii*'s antifungal activities against *C. albicans*. Using a time-kill assay and a hole-plate diffusion technique, *L. plantarum* and *L. johnsonii* cells were cultured in the presence and absence of selenium dioxide, and their cell-free spent culture medium was evaluated for antifungal activity against *C. albicans* ATCC 14053. Reduced selenium dioxide to elemental selenium nanoparticles associated with cells of both *L. plantarum* and *L. johnsonii*. Excessive antifungal activity against *C. albicans* was seen in the cell-free spent culture medium of both *Lactobacillus* species after 48 hours of growth in selenium dioxide. Additionally, cultures treated with selenium dioxide showed enhanced cell biomass antifungal efficacy against *C. albicans*. In aged people, it can help encourage the development of helpful bacteria, which inhibits the growth of harmful bacteria and, as a result, lessens the activation of pro-inflammatory cytokines (IL-6, IL-1β, and TNFa). As a result, there is less chance of chronic inflammation forming, boosting phagocytic and NK cell activity to encourage the elimination of pathogens and raising IL-10 levels, an anti-inflammatory cytokine, to combat chronic inflammation.

Meat provides essential amino acids, minerals, and B vitamins, supporting muscle synthesis in older adults. However, tough texture and reduced chewing efficiency hinder digestion. To enhance protein absorption, meat products with modified textures and pretreatment methods are needed. In an extensive review, Lee et al. have discussed the role of probiotics in enhancing protein digestion in elderly people [121]. Probiotics, such as *Lactobacillus *and *Bacillus* strains, improve protein digestibility by enhancing digestive enzyme activity and remodeling gut microbiota. Moreover, probiotics boost protein metabolism and nutrient uptake, making them a promising strategy to support digestion in aging individuals, adding them to the healthcare professional's recommendations list.

Reasons for Limited Literature

After a thorough review of the available literature, we have found that clinical studies specifically focusing on the use of nanoparticle-encapsulated probiotics in the elderly population are limited. While there is ongoing research into the benefits of probiotics for older adults and advancements in nanoparticle encapsulation techniques to enhance probiotic efficacy, clinical trials combining these two areas, particularly in geriatric subjects, are scarce. For instance, a systematic meta-analysis evaluated 10 studies on nanoparticle-mediated probiotic delivery but did not specifically address elderly populations [122].

Similarly, a clinical trial investigated the efficacy of probiotics in elderly patients undergoing orthopedic surgery to reduce postoperative cognitive dysfunction [123]. However, this study did not utilize nanoparticle-encapsulated probiotics. Given the current lack of targeted clinical studies in this area, further research is needed to explore the potential benefits and safety of nanoparticle-encapsulated probiotics in the geriatric population.

## Conclusions

Nanotechnology enables the development of bioactive nanostructures capable of enhancing the functionality of probiotics. Nanomaterials may serve as carriers for probiotic-derived bioactive compounds, facilitating their targeted delivery and enhancing their bioavailability. Additionally, nanoscale formulations may promote interactions between probiotics and host cells, augmenting their therapeutic effects and fostering symbiotic relationships within the gut microbiota. Furthermore, the integration of probiotics and nanotechnology holds promise for personalized geriatric healthcare. By leveraging advances in nanoscale diagnostics and monitoring technologies, healthcare providers can assess individual microbiome profiles and tailor probiotic interventions accordingly. Nanotechnology-enabled sensors may track physiological parameters and microbial activity in real time, enabling timely adjustments to probiotic regimens based on personalized health data.

The synergistic combination of probiotics and advanced nanotechnology presents a transformative approach to enhancing geriatric health. By overcoming barriers to probiotic efficacy and enabling precise therapeutic interventions, this innovative strategy holds immense potential for addressing age-related health challenges and improving the quality of life for the growing geriatric population. However, further research is warranted to elucidate the safety, efficacy, and long-term effects of probiotic-nanotechnology hybrids in geriatric healthcare setups.
